# Fabrication of Closed Hollow Bulb Obturator Using Thermoplastic Resin Material

**DOI:** 10.1155/2015/504561

**Published:** 2015-09-27

**Authors:** Bidhan Shrestha, E. Richard Hughes, Raj Kumar Singh, Pramita Suwal, Prakash Kumar Parajuli, Pragya Shrestha, Arati Sharma, Galav Adhikari

**Affiliations:** ^1^Department of Prosthodontics & Crown and Bridge, College of Dental Surgery, BPKIHS, Dharan, Nepal; ^2^Sterling Office, 46440 Benedict Drive No. 201, Sterling, VA 20164, USA

## Abstract

*Purpose. *Closed hollow bulb obturators are used for the rehabilitation of postmaxillectomy patients. However, the time consuming process, complexity of fabrication, water leakage, and discoloration are notable disadvantages of this technique. This paper describes a clinical report of fabricating closed hollow bulb obturator using a single flask and one time processing method for an acquired maxillary defect. Hard thermoplastic resin sheet has been used for the fabrication of hollow bulb part of the obturator. *Method.* After fabrication of master cast conventionally, bulb and lid part of the defect were formed separately and joined by autopolymerizing acrylic resin to form one sized smaller hollow body. During packing procedure, the defect area was loaded with heat polymerizing acrylic resin and then previously fabricated smaller hollow body was adapted over it. The whole area was then loaded with heat cure acrylic. Further processes were carried out conventionally. *Conclusion.* This technique uses single flask which reduces laboratory time and makes the procedure simple. The thickness of hollow bulb can be controlled and light weight closed hollow bulb prosthesis can be fabricated. It also minimizes the disadvantages of closed hollow bulb obturator such as water leakage, bacterial infection, and discoloration.

## 1. Introduction

Congenital or acquired tissue defects of the palate and/or contiguous structures need special prosthesis for proper sealing [1]. The Glossary of Prosthodontic Terms defines an obturator as “a maxillofacial prosthesis used to close a congenital or acquired tissue opening, primarily of the hard palate and/or contiguous alveolar/soft tissue structures” [2]. On the basis of extent of involvement of the defects, this prosthesis may differ in shape and size. Ideally, this prosthesis should be constructed easily, be lightweight, provide better retention, support, and stability, and be functionally acceptable to the patient [3].

The obturator prosthesis plays a very important role in the functional recovery of postmaxillectomy patients [4]. For definitive palatal obturators, the undesirable weight of the prosthesis becomes a challenge as it affects the retention, stability, and support of this maxillofacial prosthesis. These difficulties lead to traumatic functional occlusion and unacceptable oroantral or oronasal seal [5]. To fabricate a lightweight prosthesis, an open hollow obturator or a closed hollow obturator is usually chosen [6].

There are many methods available to fabricate open or closed hollow bulb obturators [7]. The reduced weight of both types of prostheses makes them more readily acceptable to the patients [8]. The open hollow bulb obturator is easier to fabricate and adjust; thus it is constructed more frequently than the closed hollow obturator. However, it is difficult to polish and clean the open hollow bulb obturator which may lead to accumulation of food and nasal secretions inside the hollow part [7]. This in turn leads to malodor, an increase in weight, and chances of infection.

This paper presents a simplified technique for fabrication of closed hollow bulb obturator using thermoplastic resin material.

## 2. Case Report

**Figure 1:**
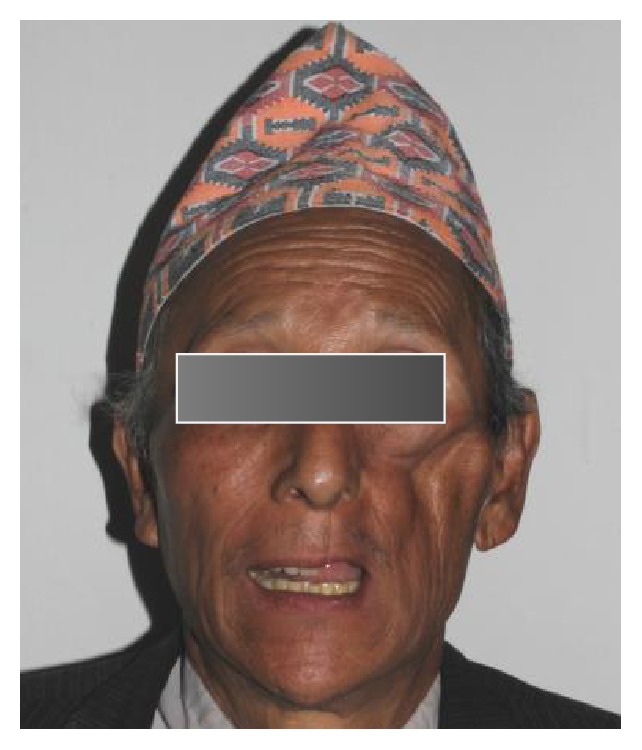
Extraoral examination.

**Figure 2:**
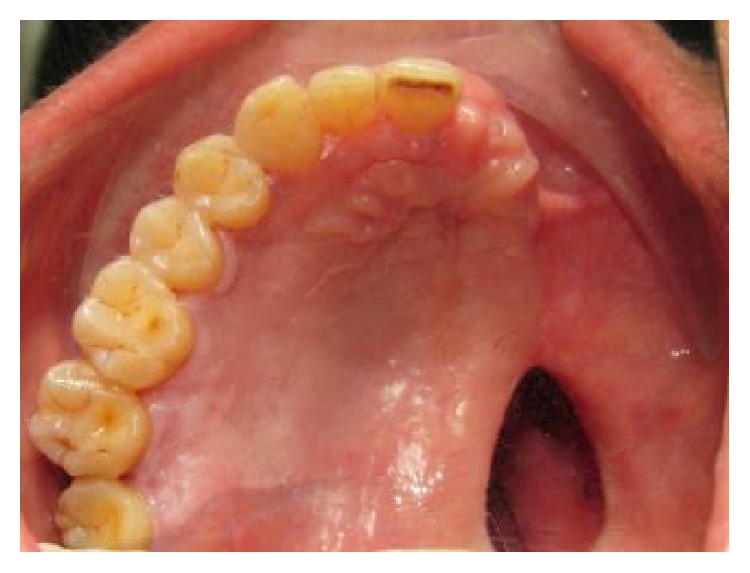
Intraoral examination of defect area.

A 67-year-old male patient was referred from the Department of Otorhinolaryngology to the Department of Prosthodontics for prosthetic rehabilitation of a postmaxillectomy case after surgical removal of squamous cell carcinoma from the left maxilla. The patient complained of difficulty in chewing, nasal regurgitation of fluids, compromised esthetics, disharmony, and difficulty in speech with nasal twang in his voice. Extraoral examination revealed gross facial asymmetry with depressed left malar region (Figure 1). Intraorally, healthy postmaxillectomy defect on the left side of maxillary edentulous area involving part of posterior hard palate, alveolar ridge, maxillary tuberosity, and some part of soft palate with intact dentition on right side with all teeth present in mandibular arch was seen (Figure 2). A hollow bulb obturator using thermoforming unit was planned for the prosthetic rehabilitation of this patient.

## 3. Procedure

**Figure 3:**
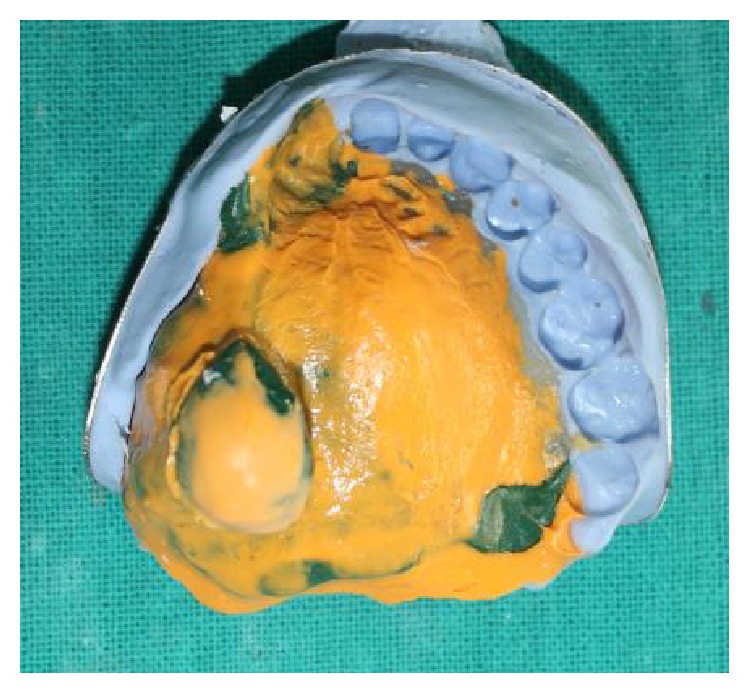
Final impression of maxillary arch.

**Figure 4:**
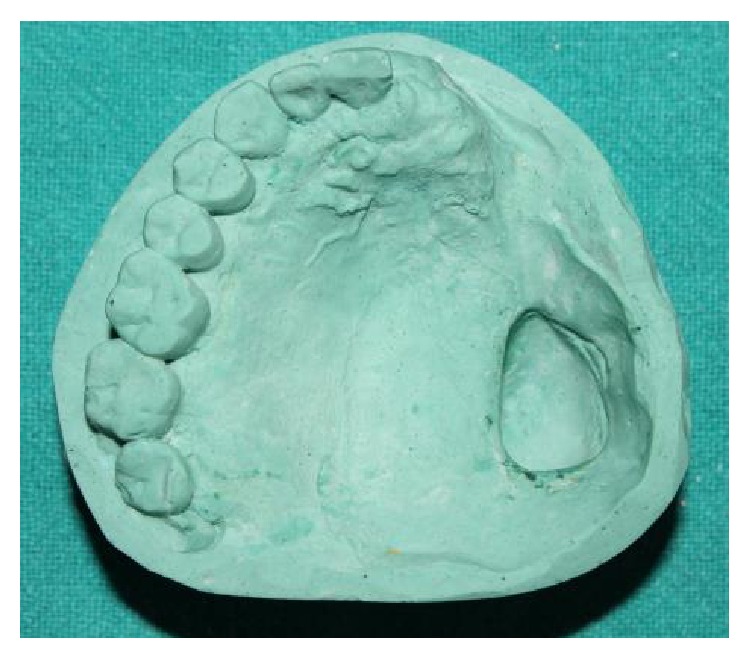
Master cast of defect area.

A preliminary impression was made using irreversible hydrocolloid (Zelgan 2002 dust-free easy mixing, DENTSPLY India Pvt. Ltd., Haryana). The custom tray was fabricated using autopolymerizing acrylic resin (self-cure acrylic repair material, DENTSPLY India Pvt. Ltd., India), and border molding was carried out using green stick impression compound (DPI Pinnacle, tracing stick, Dental Products of India, Mumbai). Final impression was made with light viscosity addition silicone impression material (Reprosil, DENTSPLY Caulk, USA) as shown in Figure 3 and the master cast was fabricated using dental stone (Kalstone, Kalabhai Karson Pvt. Ltd., India) (Figure 4). The undesirable undercuts present in the defect were blocked out in wax.

**Figure 5:**
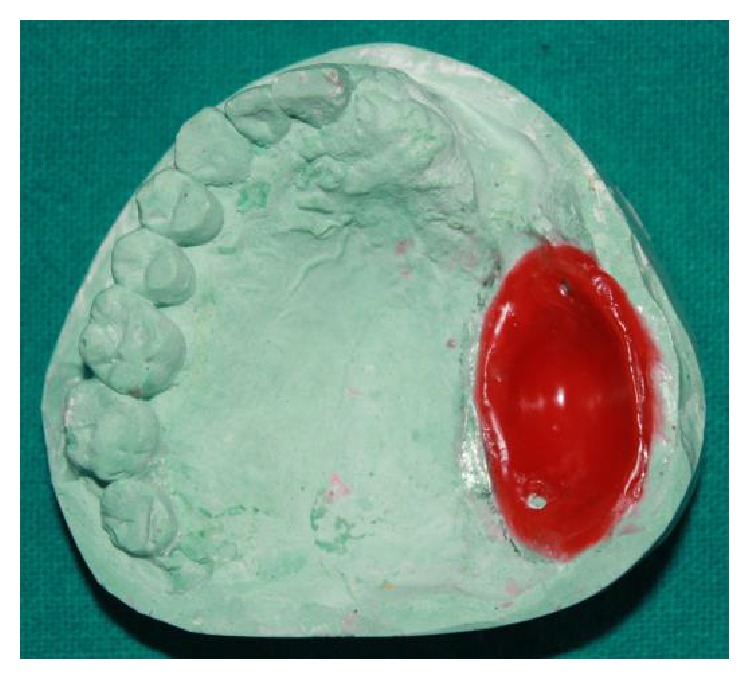
Adaptation of baseplate wax in the defect with stoppers for relief.

Base plate wax of 1.5 mm thickness was adapted in the defect for relief. Several dimples were made as stoppers for positioning in the defect (Figure 5). Thermoplastic resin 1.5 mm thick (Erkodent) was used to fabricate the bulb part of the obturator in a thermoforming unit (Erkopress 300Tp).

**Figure 6:**
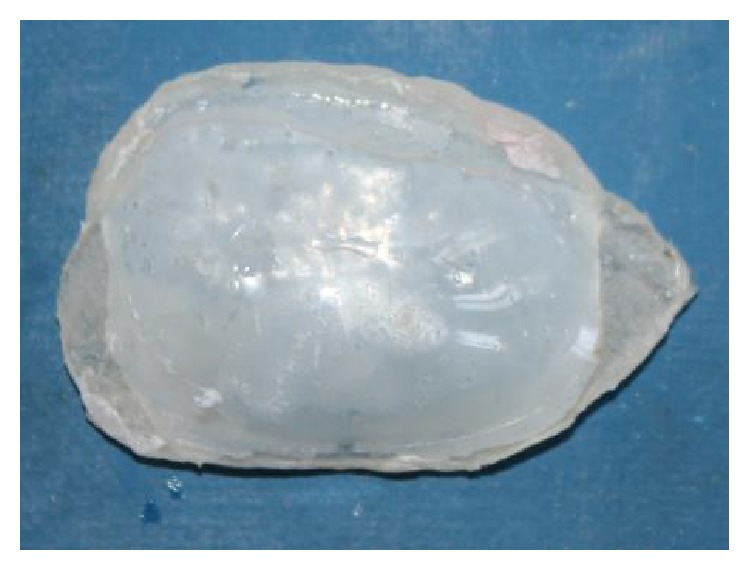
Bulb template of thermoplastic resin material.

The lid part of the obturator was then fabricated with thermoplastic resin (Erkodent). The bulb part and lid part were bonded with autopolymerizing resin to obtain a one-size smaller hollow body (Figure 6). The tight seal was confirmed by immersing it in water.

**Figure 7:**
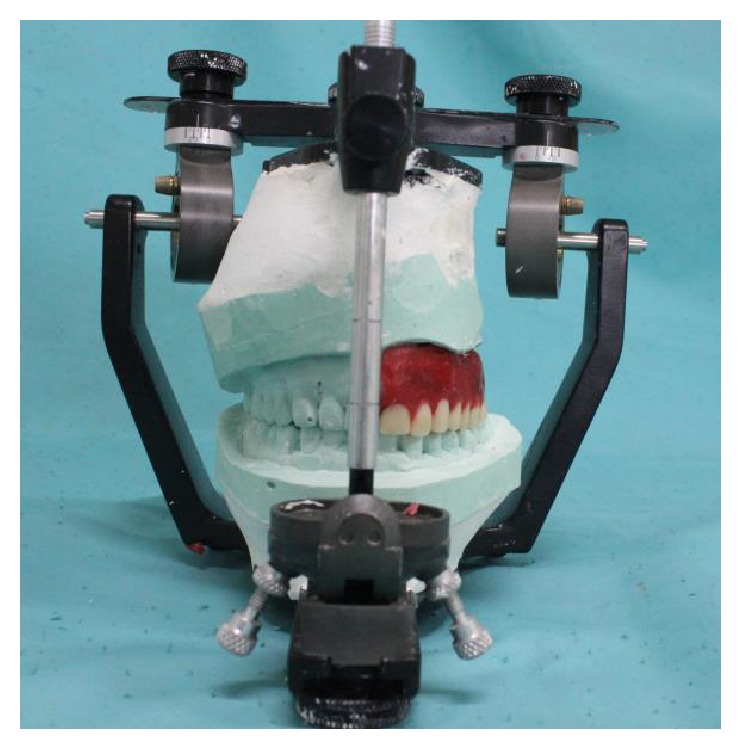
Articulation and arrangement of teeth in Hanau Wide Vue Articulator.

Using autopolymerizing resin, a denture record base was prepared and wax occlusal rim was made over it. Maxillomandibular relationship was then recorded and transferred to a semiadjustable articulator (Hanau Wide Vue Articulator). Selection and arrangement of teeth were done (Figure 7) and try-in was performed on the patient for retention, stability, function, and esthetics.

**Figure 8:**
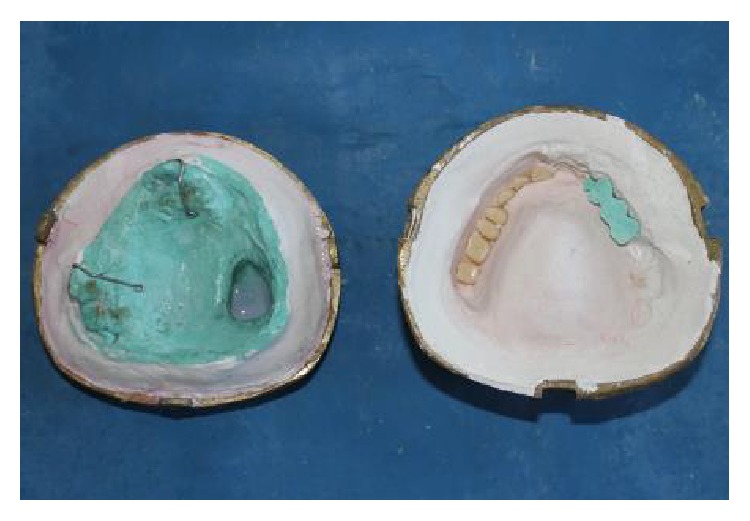
After dewaxing process.

**Figure 9:**
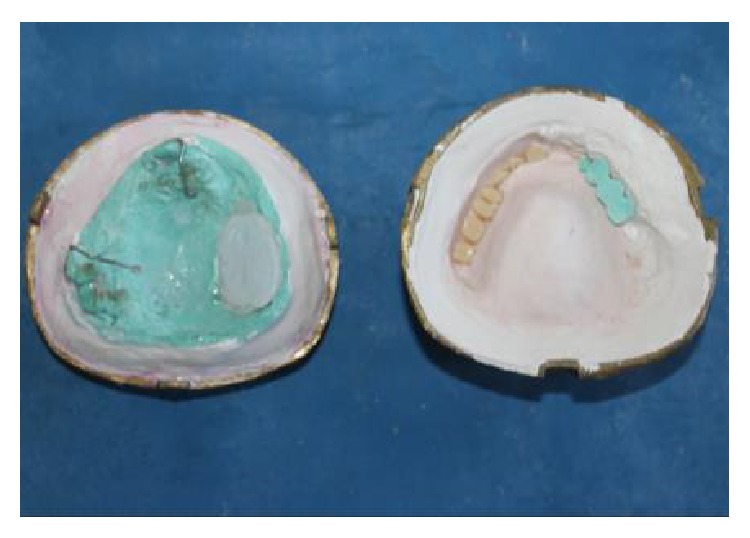
Adaptation of bulb template in defect area before packing.

**Figure 10:**
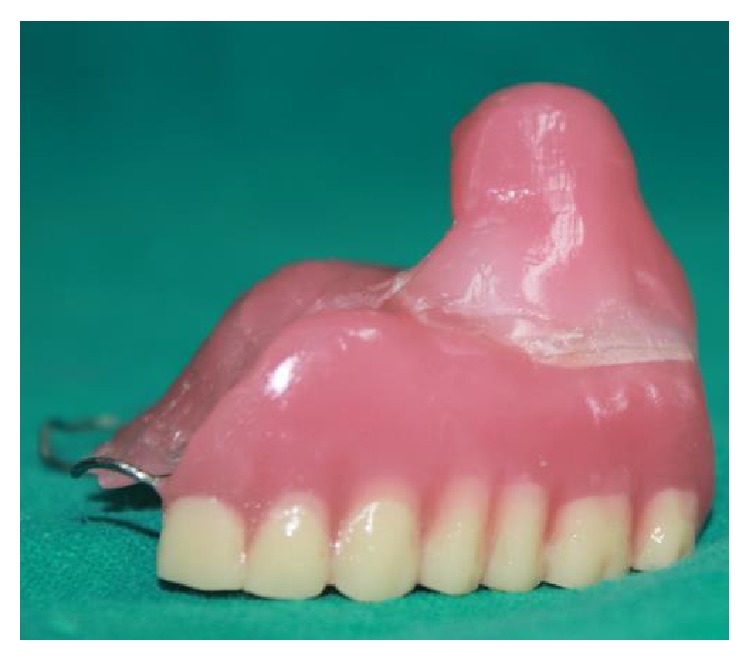
Final prosthesis.

**Figure 11:**
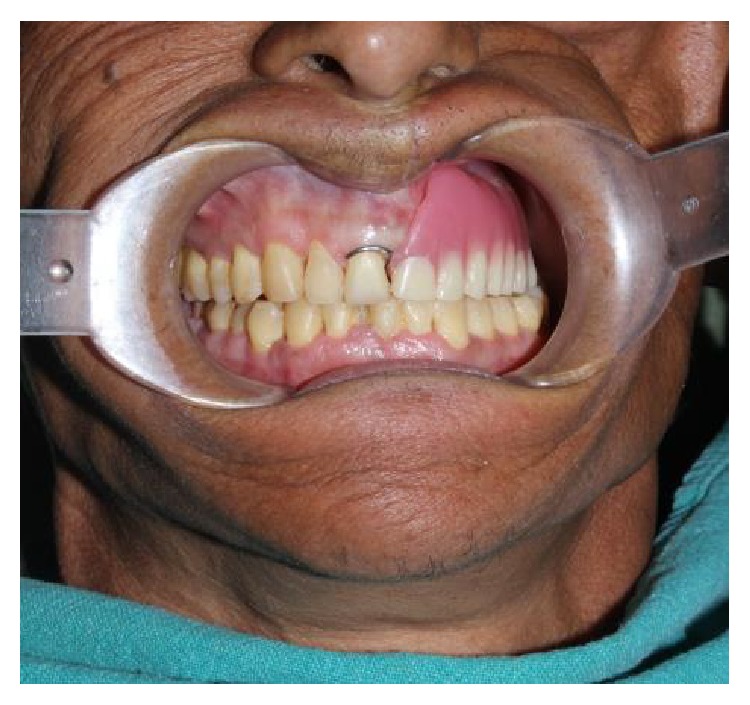
Final prosthesis in situ.

**Figure 12:**
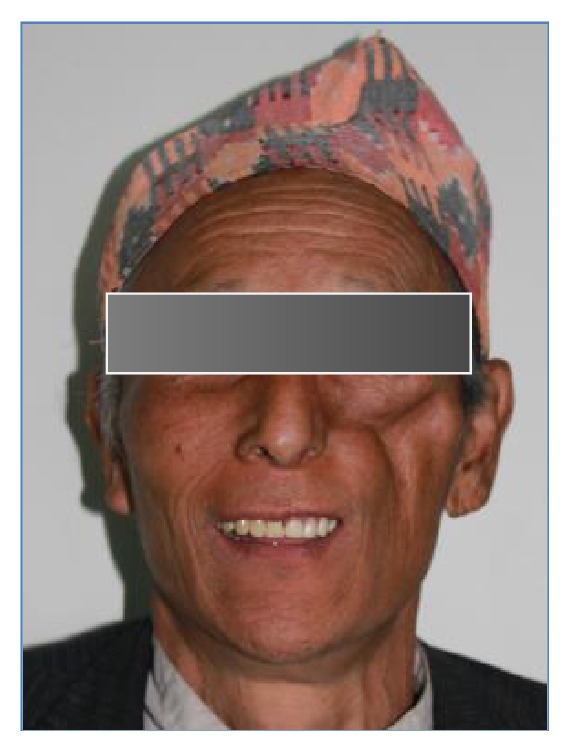
Smile view of patient after wearing prosthesis.

After successful try-in, flasking and dewaxing procedures were carried out (Figure 8). During the packing procedure, the defect area was first loaded and packed with heat polymerizing acrylic resin (Trevalon Denture Material, DENTSPLY India Pvt. Ltd., India), and then the previously fabricated smaller hollow body was adapted over it (Figure 9). Finally, the whole area was loaded with heat cure acrylic and curing was done. Finishing and polishing procedures were carried out in a conventional manner (Figure 10). Finally, the obturator prosthesis was inserted in the patient mouth (Figure 11). Patient was happy and satisfied with his improved function, speech, and esthetics (Figure 12).

## 4. Discussion

There are several methods available in the literature for fabrication of a hollow bulb obturator that discuss the difficulty during processing of hollow bulb obturators. Habib and Driscoll described a simple method by grinding out unwanted part directly after packing [9]. However, it is hard to control the adequate thickness of the obturator and also time consuming. There are methods which fabricate a hollow bulb by using materials inside the bulb part during initial stage and allowing those materials to escape through holes in the final stage [10, 11]. These techniques simply fabricate open hollow bulbs which need a separate lid to close the bulb [12]. The sealing of the opening or joining of the bulb and lid part is done by autopolymerizing resin [1, 9] or light polymerized resin [13]. The sealed area presents as a site for water leakage, promoting bacterial growth, discoloration, and increased weight of the prosthesis [1, 14]. The method described in this report solves these problems because the separately made hollow bulb and its junction are covered by heat cure acrylic during processing. In the same way, McAndrew et al. described a technique where heat cure acrylic was used to fabricate a two-part obturator prosthesis which was heat processed twice [1]. However, there may be a chance of dimensional instability with such technique. The literature shows instances where thermoplastic resin materials have been used as an immediate or interim prosthesis [8] and for fabrication of base plate of an obturator [15]. The variety in thickness of commercially available thermoplastic resin sheets makes it possible to control the thickness and weight of the hollow bulb being fabricated with this technique. The use of single flask considerably reduces the time required for fabrication and also makes the process easier to handle. However, some difficulty may arise during adaptation of preformed hollow bulb template in the defect area before packing, which may lead to a faulty final prosthesis. There might be an impact on the strength of the prosthesis during bench curing process and also on the stability to temperature during acrylization. The published literatures regarding the disadvantages of the thermoplastic resin over conventional denture base resins are limited and require further research for clarification.

## 5. Conclusion

Several merits can be attributed to the use of this technique for fabrication of closed hollow bulb obturators. The prosthesis is fabricated using a single flask which considerably reduces the laboratory time and makes the procedure simple. The thickness of the hollow bulb can be controlled and light weight closed hollow bulb prosthesis can be fabricated. The separate fabrication of a lid is not needed thereby preventing the chance of water leakage, bacterial infection, and discoloration of the prosthesis as the preformed bulb template is completely covered by heat cure acrylic resin.
